# Silencing CD36 gene expression results in the inhibition of latent-TGF-β1 activation and suppression of silica-induced lung fibrosis in the rat

**DOI:** 10.1186/1465-9921-10-36

**Published:** 2009-05-13

**Authors:** Xin Wang, Ying Chen, Lina Lv, Jie Chen

**Affiliations:** 1 Division of Pneumoconiosis, School of Public Health, China Medical University, Shenyang, PR China

## Abstract

**Background:**

The biologically active form of transforming growth factor-β1 (TGF-β1) plays a key role in the development of lung fibrosis. CD36 is involved in the transformation of latent TGF-β1 (L-TGF-β1) to active TGF-β1. To clarify the role of CD36 in the development of silica-induced lung fibrosis, a rat silicosis model was used to observe both the inhibition of L-TGF-β1 activation and the antifibrotic effect obtained by lentiviral vector silencing of CD36 expression.

**Methods:**

The rat silicosis model was induced by intratracheal injection of 10 mg silica per rat and CD36 expression was silenced by administration of a lentiviral vector (Lv-shCD36). The inhibition of L-TGF-β1 activation was examined using a CCL-64 mink lung epithelial growth inhibition assay, while determination of hydroxyproline content along with pathological and immunohistochemical examinations were used for observation of the inhibition of silica-induced lung fibrosis.

**Results:**

The lentiviral vector (Lv-shCD36) silenced expression of CD36 in alveolar macrophages (AMs) obtained from bronchoalveolar lavage fluid (BALF) and the activation of L-TGF-β1 in the BALF was inhibited by Lv-shCD36. The hydroxyproline content of silica+Lv-shCD36 treated groups was significantly lower than in other experimental groups. The degree of fibrosis in the silica+Lv-shCD36-treated groups was less than observed in other experimental groups. The expression of collagen I and III in the silica+Lv-shCD36-treated group was significantly lower than in the other experimental groups.

**Conclusion:**

These results indicate that silencing expression of CD36 can result in the inhibition of L-TGF-β1 activation in a rat silicosis model, thus further preventing the development of silica-induced lung fibrosis.

## Background

Silicosis is a form of occupational lung disease caused by inhalation of crystalline silica dust. The pathogenesis of silicosis involves alveolar cell injury and activation followed by cytokine signaling and cell recruitment in the areas of silica dust deposition [1,2]. The cytokine transforming growth factor-β1 (TGF-β1) plays a critical role in the progression of lung fibrosis [3-6], and it has been widely studied with respect to its vital role in the development of fibrosis after injury to the lung [7-10].

TGF-β1 is synthesized by virtually all cell types in an inactive form referred to as latent TGF-β1 (L-TGF-β1) consisting of the mature TGF-β1 and latent-associated peptide (LAP). Due to the noncovalently association of mature TGF-β1 with LAP, the L-TGF-β1 is unable to be recognized by cell-surface receptors and to trigger biological responses [5,7,8]. In fact, one of the primary mechanisms of TGF-β1 regulation is the control of its conversion from a latent precursor to the biologically active form [11]. CD36, as a receptor of thrombospondin-1 (TSP-1), plays an important role in the processes of L-TGF-β1 activation. A number of studies have demonstrated that L-TGF-β1 associates with TSP-1 to form the TSP-1/L-TGF-β1 complex via the specific interaction between LAP and TSP-1. The TSP-1/L-TGF-β1 complex associates with CD36 on cell surface via the specific interaction between the YRVRFLAKENVTQDAEDNC (93–110) sequence of CD36 and the sequence CSVTCG (447–452) of TSP-1. Then, L-TGF-β1 is held at the cell surface by a TSP-1/CD36 interaction and is processed by plasmin generated by activated alveolar macrophages to produce active TGF-β1. The CD36-TSP-1/L-TGF-β1 interaction appears critical to the activation process [12,13]. We presume that silencing expression of CD36 could inhibit activation of L-TGF-β1 and result in prevention of the development of lung fibrosis.

A lentiviral vector expressing short hairpin RNA (shRNA) specific for rat CD36 (Lv-shCD36) was constructed and shown to suppress expression of CD36 and inhibit the activation of L-TGF-β1 in a rat alveolar macrophage cell line called NR8383 (data not shown and will be presented in another manuscript). In the current study, a rat silicosis model was generated by intratracheal instillation, and the inhibitory effects of Lv-shCD36 on the activation of L-TGF-β1 and the resulting antifibrotic effects were examined.

## Methods

### Experimental animals and design

Equal proportions of male and female Wistar rats at 9 weeks of age, weighing 220–240 g, were obtained from the Center of Experimental Animals, China Medical University (Shenyang, China) with a National Animal Use License number of SCXK-LN 2003-0009. The animals were housed at an environmental temperature of 24 ± 1°C and a 12/12 h light/dark cycles, with free access to food and water. SiO_2 _was purchased from Sigma (St., Louis, MO, USA). The silica content of the SiO_2 _was >99%, the dust particle size was 0.5–10 μm, and 80% of the particles were 1–5 μm. Lv-shCD36, a lentiviral vector expressing shRNA specific against rat CD36, was developed for a prior study, and it suppressed the expression of CD36 (data not shown and will be presented in another manuscript). All experiments and surgical procedures were approved by the Animal Care and Use Committee at the China Medical University, which complies with the National Institute of Health Guide for the Care and Use of Laboratory Animals.

Animals were divided randomly into the following four experimental groups (n = 24 per group): (1) saline control group: instillation of 0.5 ml sterile physiological saline; (2) silica group: instillation of a suspension of 10 mg silica dust in a total volume of 0.5 ml sterile physiological saline; (3) silica+Lv-shCD36 group: instillation of a mixed suspension of 10 mg silica dust and 5 × 10^8 ^transducing units (TU) of Lv-shCD36 in a total volume of 0.5 ml sterile physiological saline; (4) silica+Lv-shCD36-NC group: instillation of a mixed suspension of 10 mg silica dust and 5 × 10^8^TU Lv-shCD36-NC(non-silence control lentivirus) in a total volume of 0.5 ml sterile physiological saline. Rats were anesthetized with an intraperitoneal injection of 10 mg/rat pentobarbital sodium. The skin of the neck was opened and blunt dissection exposed the trachea. Either physiological saline, silica in physiological saline, or silica with Lv-shCD36 or Lv-shCD36-NC in physiological saline, was instilled into the lungs using a 14-gauge needle inserted into the trachea through the epiglottis of the larynx. The site of surgery was sutured and the rats were allowed to recover until they were sacrificed. At 7, 21 and 28 days post-instillation, eight rats of each group were anesthetized with anesthetic ether, sacrificed by decapitation, and the lungs were removed. Bronchoalveolar lavage fluid (BALF) was obtained by cannulating the trachea, injecting and retrieving 3 ml aliquots of sterile physiological saline that was centrifuged at 1000 rpm for 1 min at 4°C. The cells were incubated in 1640 medium for 2 h at 37°C in 5% CO_2_, and the adherent cells were mostly alveolar macrophages (AMs). After detection of green fluorescent protein (GFP) by fluorescence microscopy, AMs were collected for real-time PCR analysis. The BALF supernatant was centrifuged at 3000 rpm for 10 min at 4°C, and stored at -80°C for later determination of TGF-β1.

### Quantitative real-time PCR analysis

Total RNA was isolated from AMs using the Trizol reagent (Invitrogen, Carlsbad, CA, USA) according to the manufacturer's protocol. The sequences of primers specific for CD36 (sense: 5'-GAAGCACTGAAGAATCTGAAGAG-3'; antisense: 5'-TCCAACACCAAGTAAGACCATC-3'), and β-Actin (sense: 5'-CGGCATTGTCACCAACTG-3'; antisense: 5'-CGCTCGGTCAGGATCTTC-3'), were synthesized by Genechem (China). Real-time quantitative PCR (qRT-PCR) analysis was performed as previously described. Each PCR reaction mixture (20 μl) contained 10 μl of 2 × SYBR Green Master Mix (Takara, Japan), 1 μl of forward and reverse primers (5 μmol/μl), 1 μl of cDNA product and water. The PCR reactions were run on iQ5 (Bio-Rad) using the following program: 95°C for 15 s, and 40 cycles of 95°C for 5 s and 60°C for 30 s. Following PCR amplification, the reactions were subjected to a temperature ramp to create a dissociation curve, measured as change in fluorescence as a function of temperature, which allows detection of non-specific products. qRT-PCR data were analyzed using the two standard curve method and β-Actin was used as an internal control to normalize gene expression level.

### CCL-64 mink lung epithelial growth inhibition assay for TGF-β1

The CCL-64 cell line was grown in Dulbecco's Modified Eagle's Medium (DMEM, Gibco, USA) with 10% fetal bovine serum (FBS, Gibco, USA) at 37°C, in 5% CO_2_. To detect quantities of TGF-β1 in BALF, CCL-64 cells were plated at 5 × 10^3 ^cells/well in 96-well plates and cultured in FBS-free DMEM at 37°C in 5% CO_2 _for 4 h. Ten μl of untreated sample, equivalent to the quantity of TGF-β1 representing active TGF-β1, or treated samples acidified with HCl and subsequently neutralized with NaOH which were equivalent to the quantity of TGF-β1 representing the total TGF-β1 of the same sample, were added to the wells. The standard curve contained concentrations ranging from 31.25 to 2000 pg/ml of porcine TGF-β1 (R&D Systems, Minneapolis, USA). The plates were incubated at 37°C in 5% CO_2 _for 24 h, then added 10 μl MTT reagent (5 mg/ml final concentration) to each well for 4 h of incubation. The plates were added 100 μl DMSO to dissolve the precipitate before analysis at 570 nm using a microplate reader (Bio-Rad 550) [14].

### Determination of hydroxyproline content

The lung samples were measured for hydroxyproline content using a hydroxyproline kit from Nanjing Jian Cheng Institute (China) following instructions of the manufacturer. The results were calculated as micrograms of hydroxyproline per gram of wet lung weight using hydroxyproline standards.

### Pathological examination

Following gross inspection of each mouse, small pieces of lung tissue from the middle of the lobes, in addition to the hilar lymph nodes, were fixed with 4% paraformaldehyde, embedded in paraffin, and sectioned at 5 μm. The tissue sections were stained with hematoxylin and eosin (HE) and van Gieson's stain (vG) for collagen fibers. Silicotic nodules were graded as following: cellular nodules as Stage I; fibrotic cellular nodules as Stage II; cellular fibrotic nodules as Stage III; fibrotic nodules as Stage IV.

### Immunohistochemical staining

For immunohistochemical examination, all sections were deparaffinized in xylene followed by 100% ethanol and then placed in a freshly prepared methanol plus 3% H_2_O_2 _solution for 30 min to block endogenous peroxidase activity. After overnight incubation at 4°C with rabbit polyclonal anti-collagen I and III antibodies (Santa Cruz Biotechnology, Santa Cruz, CA, USA) diluted 1:100 in phosphate-buffered saline (PBS), antigen-antibody complexes were detected using Streptavidin/Peroxidase (SP) Histostain™-Plus Kits (Beijing Zhongshan Golden Bridge Biotechnology Ltd., China). Peroxidase activity was revealed using a 3, 3'-diaminobenzidine tetrahydrochloride Substrate Kit (Beijing Zhongshan Golden Bridge Biotechnology Ltd., China). The sections were counterstained with hematoxylin for 3 min, rinsed and mounted with glycerin gelatin for histological examination. Brown particles in the cytoplasm or the cellular membrane were considered a positive reaction. The collagen I and III proteins were analyzed quantitatively using MetaMorph/DP10/BX41-type image analysis software (UIC/OLYMPUS, US/JP). In 10 × 40 fields, three to five fields were randomly selected for each section. The integrated optical density (IOD) average represented the quantitative expression of collagens I and III.

### Statistical analyses

SPSS 13.0 software was used to conduct statistical analyses. The differences between values were evaluated through one-way analysis of variance (ANOVA) followed by pair-wise comparison with the Student-Newman-Keuls test. P < 0.05 was considered statistically significant.

## Results

### Lv-shCD36 could silence the expression of CD36 in AMs

**Figure 1:**
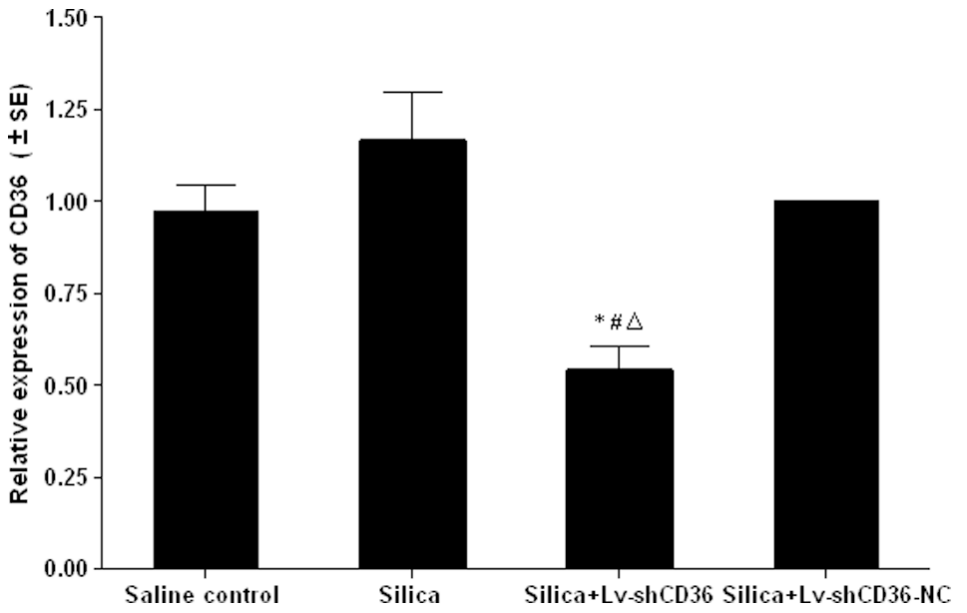
**CD36 mRNA levels from the AMs of each group were detected by realtime-PCR**. The expression of CD36 mRNA in the silica+Lv-shCD36 group was significantly lower than in the saline control, silica, or silica+Lv-shCD36-NC groups at 7 days. Each bar represents the mean ± SEM. *P < 0.05, as compared to saline control group;^Δ^*P *< 0.05, as compared to silica group; and ^#^*P *< 0.05, as compared to the silica+Lv-shCD36-NC group. Data was repeated twice (n = 3) and similar results were obtained.

AMs were obtained from BALF in each experimental group at 7 days after instillation. The AMs infected with either Lv-shCD36 or Lv-shCD36-NC expressed GFP fluorescence, which was detected by fluorescent microscope [see Additional file 1]. Real-time PCR was performed to determine the silencing effect of CD36 in AMs in the silica+Lv-shCD36 group. The results demonstrate that expression of CD36 mRNA in the silica+Lv-shCD36 group was significantly lower than in the saline control, silica, and silica+Lv-shCD36-NC groups (P < 0.05) at 7 days (Figure 1).

### Inhibition of L-TGF-β1 activation by Lv-shCD36 in BALF

**Figure 2:**
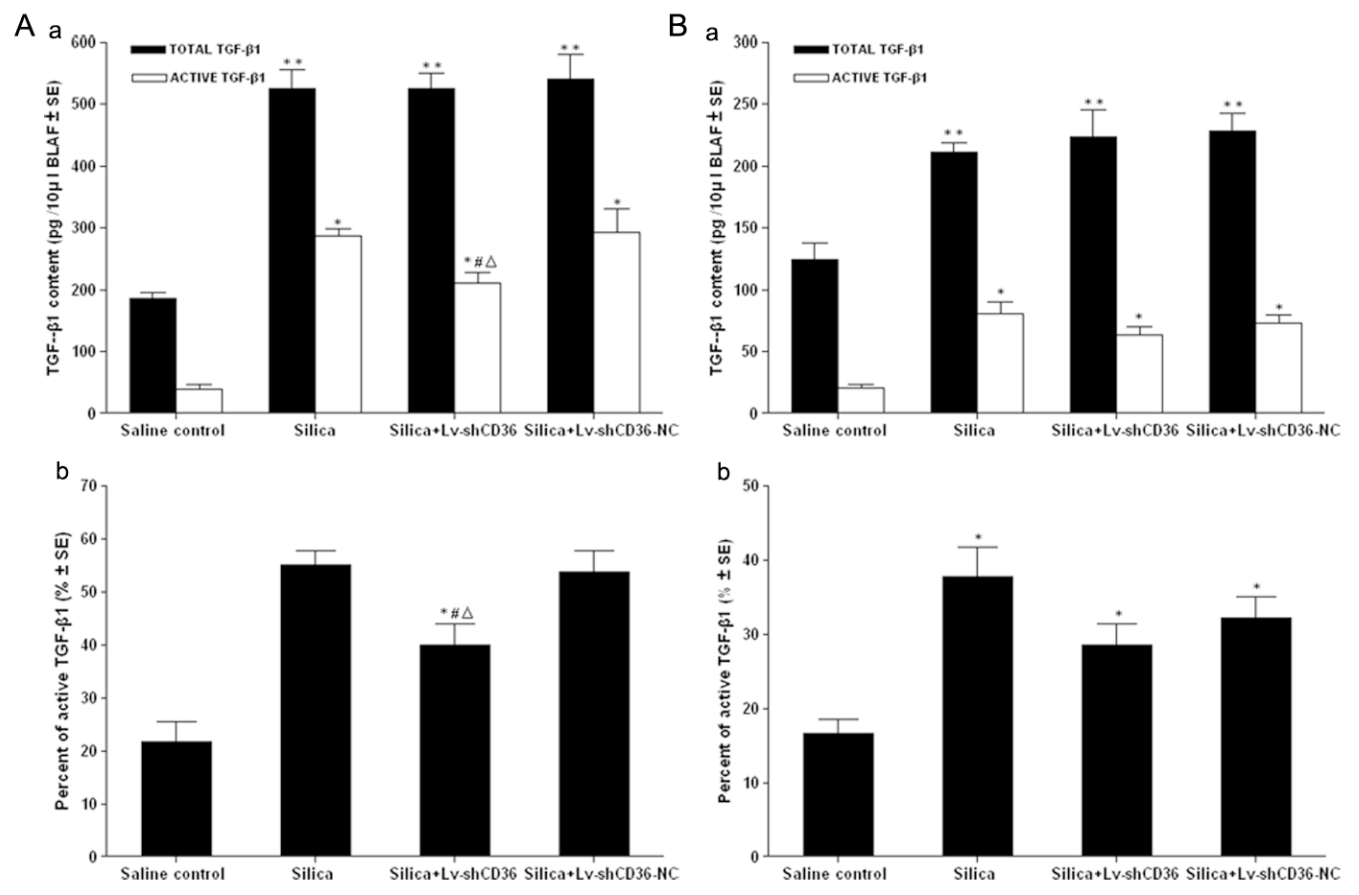
**Quantity of TGF-β1 and the percentage of active TGF-β1 in BALF**. **(A–a) **The quantity of total and active TGF-β1 in the silica, silica+Lv-shCD36, and silica+Lv-shCD36-NC groups were significantly higher than in the saline control group at 7 days after instillation. The quantity of active TGF-β1 in the silica+Lv-shCD36 group was significantly lower than in the silica and silica+Lv-shCD36-NC groups at 7 days. **(A–b) **The percentage of active TGF-β1 in the silica+Lv-shCD36 group was significantly lower than in the silica and silica+Lv-shCD36-NC groups, and it was significantly higher than in the saline control group at 7 days after instillation. **(B–a) **The quantity of total and active TGF-β1 in the silica group, the silica+Lv-shCD36 group and the silica+Lv-shCD36-NC group were significantly higher than in the saline control group at 21 days after instillation. **(B–b) **The percentage of active TGF-β1 in the silica group, the silica+Lv-shCD36 group and the silica+Lv-shCD36-NC group were significantly higher than in the saline control group at 21 days after instillation. Each bar represents the mean ± SEM. **P < 0.05, as compared to the quantity of total TGF-β1 in the saline control group; *P < 0.05, as compared to the quantity of active TGF-β1 in the saline control group; ^Δ^*P *< 0.05, as compared to the quantity of active TGF-β1 in the silica group; ^#^*P *< 0.05, as compared to the quantity of active TGF-β1 in the silica+Lv-shCD36-NC group. The data represent the means from experiments done in six rats.

The activation of L-TGF-β1 was determined by detecting the quantity of TGF-β1 in BALF using the CCL-64 growth inhibition assay. The quantities of total TGF-β1 and active TGF-β1 from BALF in silica group, silica+Lv-shCD36 group and silica+Lv-shCD36-NC group were significantly higher than those of the saline control group (P < 0.05) at 7 days after the instillations. The quantity of active TGF-β1 from BALF in the silica+Lv-shCD36 group was significantly lower than in the silica group or the silica+Lv-shCD36-NC group (P < 0.05) (Figure 2A-a). The percent of active TGF-β1 in BALF from each sample was derived using active TGF-β1 as the numerator and total TGF-β1 as the denominator. The percent of active TGF-β1 from BALF in the silica+Lv-shCD36 group was significantly lower than in the silica group or the silica+Lv-shCD36-NC group (P < 0.05), and it was significantly higher than that of the saline control group (P < 0.05) (Figure 2A-b). At 21 days after instillation, the quantity of total TGF-β1 and active TGF-β1 from BALF in the silica group, the silica+Lv-shCD36 group and the silica+Lv-shCD36-NC group was decreased compared with the results at 7 days, and they were significantly higher than the results from the saline control group (P < 0.05). There were no significant differences among the silica group, the silica+Lv-shCD36 group and the silica+Lv-shCD36-NC group (Figure 2B).

### Lv-shCD36 could reduce hydroxyproline content in lung

**Figure 3:**
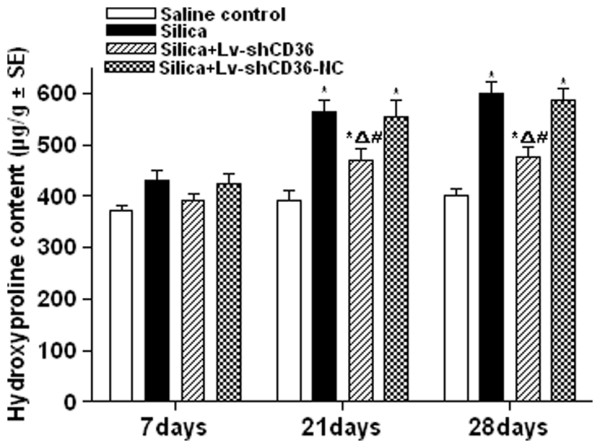
**Hydroxyproline content of rat lungs**. There was no significant difference in the hydroxyproline content of the four groups at 7 days after instillation. The hydroxyproline content of the silica, the silica+Lv-shCD36, and the silica+Lv-shCD36-NC groups was significantly higher than that of the saline control group at 21 and 28 days after instillation. The hydroxyproline content of the silica+Lv-shCD36 group was significantly lower than the silica and silica+Lv-shCD36-NC groups at 21 and 28 days after instillation. Each bar represents the mean ± SEM. *P < 0.05, as compared to the saline control group;^Δ^*P *< 0.05, as compared to the silica group; and ^#^*P *< 0.05, as compared to the silica+Lv-shCD36-NC group. The data represent the means from experiments done in eight rats.

Hydroxyproline content is an important indicator of lung fibrosis. In this study, no significant differences in the hydroxyproline content of the four treatment groups were observed at 7 days after instillation when measured using a hydroxyproline kit. However, the hydroxyproline contents of the silica group and the silica+Lv-shCD36-NC group were significantly higher than those of the saline control group (P < 0.05) at 21 and 28 days after instillation. The hydroxyproline content of the silica+Lv-shCD36 group was significantly lower than in the silica group and the silica+Lv-shCD36-NC group (P < 0.05), and significantly higher than that of the saline control group (P < 0.05) at 21 and 28 days after instillation (Figure 3).

### Lv-shCD36 could inhibit silica-induced lung fibrosis

**Figure 4:**
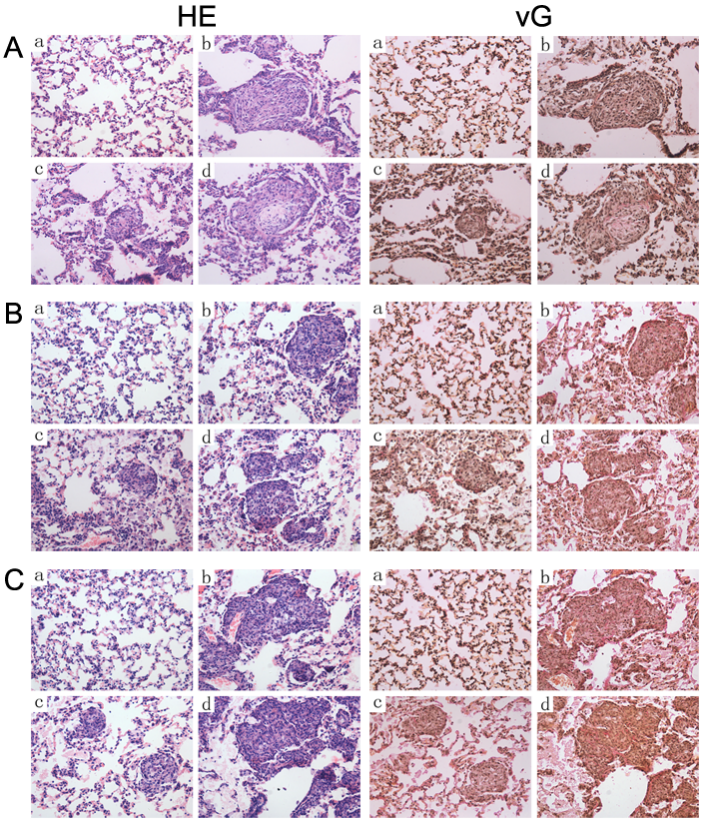
**HE and vG staining for histopathologic changes in rat lungs (× 400)**. **(A) **7 days after instillation; **(B) **21 days after instillation; **(C) **28 days after instillation. a: saline control group; b: silica group; c: silica+Lv-shCD36; and d: silica+Lv-shCD36-NC.

The lung tissues of rats were observed by light microscope to monitor pathological changes. No obvious abnormalities were observed in the lungs of rats that received physiological saline. However, in the silica and silica+Lv-shCD36-NC groups, there was a large infiltration of inflammatory cells and alveolar septal thickening in the lung, and occasionally a small amount of cellular nodules (Stage I) and tiny collagen fibers were observed, at 7 days after instillation. There were less cellular nodules (Stage I) in the lungs of rats in the silica+Lv-shCD36 group, and the vG stain was weakly positive for collagen fibers. At 21 days after instillation, primarily cellular nodules and fibrotic cellular nodules (Stage I and II) were observed in the silica and the silica+Lv-shCD36-NC groups. Some nodules arranged close and some nodules had loosely distributed collagen fibers. There were mainly cellular nodules (Stage I) and tiny collagen fibers in the lung of rats in the silica+Lv-shCD36 group. Compared to the silica and the silica+Lv-shCD36-NC groups, the number of nodules in the lungs of rats in the silica+Lv-shCD36 group was fewer, and they were smaller. In the silica and the silica+Lv-shCD36-NC groups, fibrotic cellular nodules (Stage II) and loosely distributed collagen fibers were observed at 28 days after the instillation. There were still mostly cellular nodules (Stage I) and tiny collagen fibers in the lungs of rats from the silica+Lv-shCD36 group, but the number of nodules was increased (Figure 4, Table 1).

**Table 1:** Silicatic nodule grade of the rat lungs in each group

	7 days after instillation		21 days after instillation		28 days after instillation	
			
Groups	n	Silicotic nodule grade	n	Silicotic nodule grade	n	Silicotic nodule grade
Saline control	8	0	8	0	8	0
Silica	8	I	8	I ~II	8	II
Silica +Lv-shCD36	8	0 ~I	8	I	8	I
Silica +Lv-shCD36-NC	8	I	8	I ~II	8	II

### Lv-shCD36 could inhibit the expression of the collagen I and III in lung

**Figure 5:**
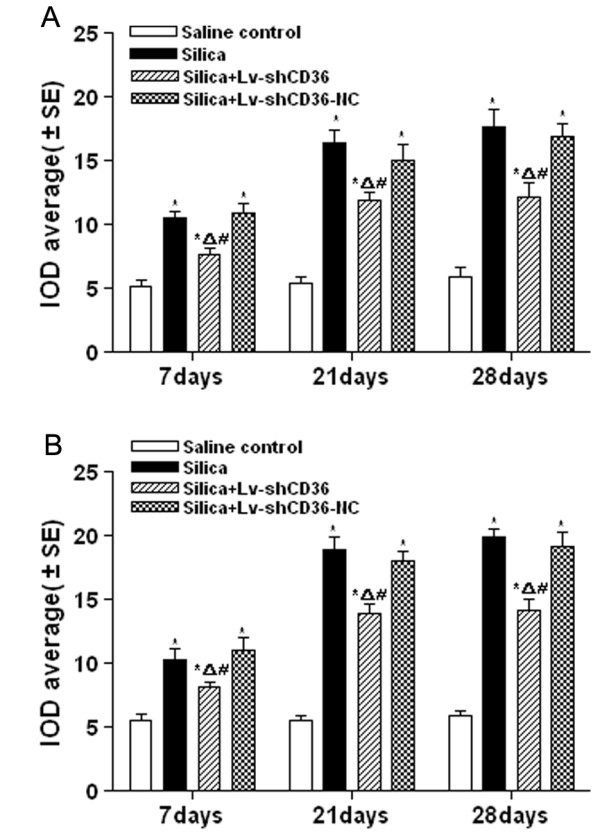
**IOD average of collagen I and III in the rat lungs**. **(A) **The IOD averages of collagen I of the silica, the silica+Lv-shCD36 and the silica+Lv-shCD36-NC groups were significantly higher than those of the saline control group at three time points. The IOD averages of collagen I of the silica+Lv-shCD36 group were significantly lower than those of the silica and the silica+Lv-shCD36-NC groups. **(B) **The IOD averages of collagen III of the silica, the silica+Lv-shCD36 and the silica+Lv-shCD36-NC groups were significantly higher than those of the saline control group at three time points. The IOD averages of collagen III of the silica+Lv-shCD36 group were significantly lower than those of the silica and the silica+Lv-shCD36-NC groups. Each bar represents the mean ± SEM. *P < 0.05, as compared to the saline control group;^Δ^*P *< 0.05, as compared to the silica group; and ^#^*P *< 0.05, as compared to the silica+Lv-shCD36-NC group. The data represent the means from experiments done in six rats.

To further observe the degree of fibrosis, immunohistochemical examination of collagen I and III was performed in the lung tissue. The results showed a weakly positive reaction for scattered collagen I and collagen III in the mesenchymal tissue of the saline control group. The expressions of collagen I and collagen III of the silica+Lv-shCD36 group were weaker than those of the silica and silica+Lv-shCD36-NC groups [see Additional file 2 and Additional file 3]. At the three time points after the instillation, the IOD average of collagen I in the silica and the silica+Lv-shCD36-NC groups was significantly higher than that of the saline control groups (P < 0.05). The IOD average of collagen I in the silica+Lv-shCD36 group was higher than that of the saline control group (P < 0.05), but was significantly lower than that of the silica and silica+Lv-shCD36-NC groups (P < 0.05) (Figure 5A). The collagen III results were concordant with those of collagen I (Figure 5B).

## Discussion

Lung fibrosis is the most important pathological change in silicosis. An experimental animal model of silicosis was induced by an intratracheal administration of silica dust, resulting in varying degrees of fibrotic silicosis [15]. Following silica-induced lung injury, AMs were stimulated and they secreted large quantities of biologically active TGF-β1, which plays a critical role in the development of lung fibrosis [16-19]. Recent research suggests that a polyclonal anti-TGF antibody or the proteoglycan decorin, a TGF-β1 binding protein, could block TGF-β1 and markedly reduce extracellular matrix accumulation [20-22]. We hypothesized that it would be also effective to inhibit the activation of TGF-β1 in an attempt to prevent development of silica-induced lung fibrosis.

CD36 may be involved in silica-induced lung fibrosis, because of its specific combination with TSP-1, which is a critical factor in the activation of L-TGF-β1 [12]. Accordingly, in our previous work RNAi technology was used to construct a recombinant lentiviral vector, Lv-shCD36, which expresses shRNA specific against rat CD36. Lv-shCD36 was demonstrated to inhibit the activation of L-TGF-β1 *in vivo *using the rat alveolar macrophage cell line NR8383 (data not shown and will be presented in another manuscript). In the current study, Lv-shCD36 was used to test for inhibition of L-TGF-β1 activation and an antifibrotic effect in a rat silicosis experimental model. To determine effect of Lv-shCD36 on CD36 in the lungs of rats, AMs were isolated from BALF seven days after instillation to detect the expression of CD36 mRNA by real time-PCR. The result suggests that Lv-shCD36 can suppress CD36 mRNA expression in the AMs. Examination of total and active TGF-β1 in the BALF in the early phase of the experimental silicosis demonstrated that Lv-shCD36 could depress the quantity and percentage of active TGF-β1. Therefore, we believe that Lv-shCD36 could inhibit activation of L-TGF-β1 by decreasing expression of CD36 on the membrane of AMs to further reduce the combination of CD36 with TSP-1/L-TGF-β1.

Activated TGF-β1 can bind its receptor on membrane of lung fibroblast to regulate collagen synthesis and degradation that ultimately results in lung fibrosis [8,17]. Inhibiting activation of L-TGF-β1 could suppress development of silica-induced lung fibrosis. This study shows that the hydroxyproline content of the silica+Lv-shCD36 group was significantly lower than the silica and silica+Lv-shCD36-NC groups at 21 and 28 days after instillation. The results of immunohistochemical examination of collagen I and III showed that the IOD average of both collagens of the silica+Lv-shCD36 group were significantly lower than in the silica and silica+Lv-shCD36-NC groups. Furthermore, the pathological examination revealed an obviously lighter degree of fibrosis in the silica+Lv-shCD36 group than in the silica and silica+Lv-shCD36-NC groups. We conclude that Lv-shCD36 could reduce pathological tissue fibrosis and collagen accumulation in the rat model of silicosis, and therefore, it could inhibit the development of silicosis.

In the experimental lung fibrosis model, the AMs generate maximal quantities of L-TGF-β1 at 7 days after instillation of the early phase of lung fibrosis. After L-TGF-β1 was processed to become active form, the active TGF-β1 starts the occurrence and development of lung fibrosis. In the mid and late phases of the experimental lung fibrosis model, the amount of TGF-β1 released by the AMs declines gradually [23,24]. In the experimental silicosis model, there are primarily inflammatory changes and cellular nodules in the early phase. With the development of fibrosis, there are fibrotic cellular nodules, cellular fibrotic nodules, even fibrotic nodules in the mid and late phases of the experimental silicosis [25]. This study also shows that in the early phase, the quantities of L-TGF-β1 were obvious high compared with those at 21 days after instillation, and the quantity and percentage of active TGF-β1 were depressed by Lv-shCD36 at 7 days after instillation. At 21 and 28 days after instillation, the degree of silicosis was inhibited obviously by Lv-shCD36. Accordingly, CD36 may participate in the activation process of L-TGF-β1 in the early phase of the experimental silicosis. Furthermore, silencing expression of CD36 prevented the development of silicosis via inhibiting the activation of L-TGF-β1. Silicosis patients usually have exposed to low dose of silica dust for a long time. Silicosis is a chronic and progressive pathologic reaction. The pathological process of silicosis occurs and develops repeatedly. So, we presume that CD36 also repeatedly participates in the activation of L-TGF-β1 and the pathological process of silicosis. These results provide a new molecular basis for understanding latency and activation of L-TGF-β1, which should aid in the design of novel strategies to suppress silica-induced lung fibrosis through modulating inappropriate levels of TGF-β1 activity.

## Conclusion

We have shown that silencing expression of CD36 inhibits activation of L-TGF-β1, which results in reduced hydroxyproline, collagen synthesis, and further prevention of the development of lung fibrosis. These effects may be through the suppression of the association of the TSP-1/L-TGF-β1 complex with CD36. Our data support the view that CD36 may contribute to the control of the activation of L-TGF-β1 and, therefore, silencing expression of CD36 could inhibit development of silica-induced lung fibrosis.

## Abbreviations

L-TGF-β1: latent transforming growth factor-β1; TSP-1: thrombospondin-1; shRNA: short hairpin RNA; BALF: bronchoalveolar lavage fluid; AMs: alveolar macrophages.

## Competing interests

The authors declare that they have no competing interests.

## Authors' contributions

XW carried out the experiments, participated in the experimental design and in the interpretation of data, and drafted the manuscript. YC conceived of the study, participated in the analysis of data, helped draft the manuscript. LL participated in the animal instillation and in the histological and immunohistochemical experiments. JC initiated the project, participated in the design of the study and in the interpretation of data, and revised the manuscript critically.

## Supplementary Material

Additional File 1 **Expression of GFP in AMs obtained from BALF (× 200)**. AMs obtained from BALF at 7 days after instillation, were assayed for GFP expression by fluorescent microscopy. a) silica+Lv-shCD36 group; b) silica+Lv-shCD36-NC group. AMs infected with either Lv-shCD36 or Lv-shCD36-NC expressed GFP fluorescence, demonstrating that the Lv-shCD36 and Lv-shCD36-NC could infect AMs successfully *in vivo*. Click here for file

Additional File 2 **Immunohistochemical staining for collagen I at each time point (× 400)**. 7d: a) saline control group, b) silica group, c) silica+Lv-shCD36, and d) silica+Lv-shCD36-NC; 21d: e) saline control group, f) silica group, g) silica+Lv-shCD36, and h) silica+Lv-shCD36-NC; 28d: i) saline control group, j) silica group, k) silica+Lv-shCD36, and l) silica+Lv-shCD36-NC. The expression of collagen I in the saline control group was negative at three time points. The expression of collagen I in the silica+Lv-shCD36 group was weaker than in the silica group and the silica+Lv-shCD36-NC group at three time points. Click here for file

Additional File 3 **Immunohistochemical staining for collagen III at each time point (× 400)**. 7d: a) saline control group, b) silica group, c) silica+Lv-shCD36, and d) silica+Lv-shCD36-NC; 21d: e) saline control group, f) silica group, g) silica+Lv-shCD36, and h) silica+Lv-shCD36-NC; 28d: i) saline control group, j) silica group, k) silica+Lv-shCD36, and l) silica+Lv-shCD36-NC. The expression of collagen I in the saline control group was negative at three time points. The expression of collagen III in the silica+Lv-shCD36 group was weaker than in the silica group and the silica+Lv-shCD36-NC group at three time points. Click here for file
