# Supplementary figures and images for: Silencing CD36 gene expression results in the inhibition of latent-TGF-β1 activation and suppression of silica-induced lung fibrosis in the rat
Source: Respir Res. 2009 May 13;10(1):36. doi: 10.1186/1465-9921-10-36 (PMC2698900; doi:10.1186/1465-9921-10-36)

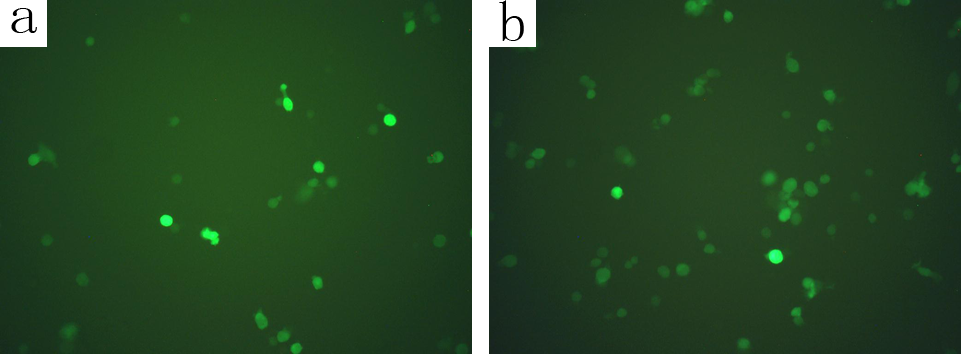

Supplement: Additional File 1 — Expression of GFP in AMs obtained from BALF (× 200). AMs obtained from BALF at 7 days after instillation, were assayed for GFP expression by fluorescent microscopy. a) silica+Lv-shCD36 group; b) silica+Lv-shCD36-NC group. AMs infected with either Lv-shCD36 or Lv-shCD36-NC expressed GFP fluorescence, demonstrating that the Lv-shCD36 and Lv-shCD36-NC could infect AMs successfully in vivo. [file 1465-9921-10-36-S1.tiff]

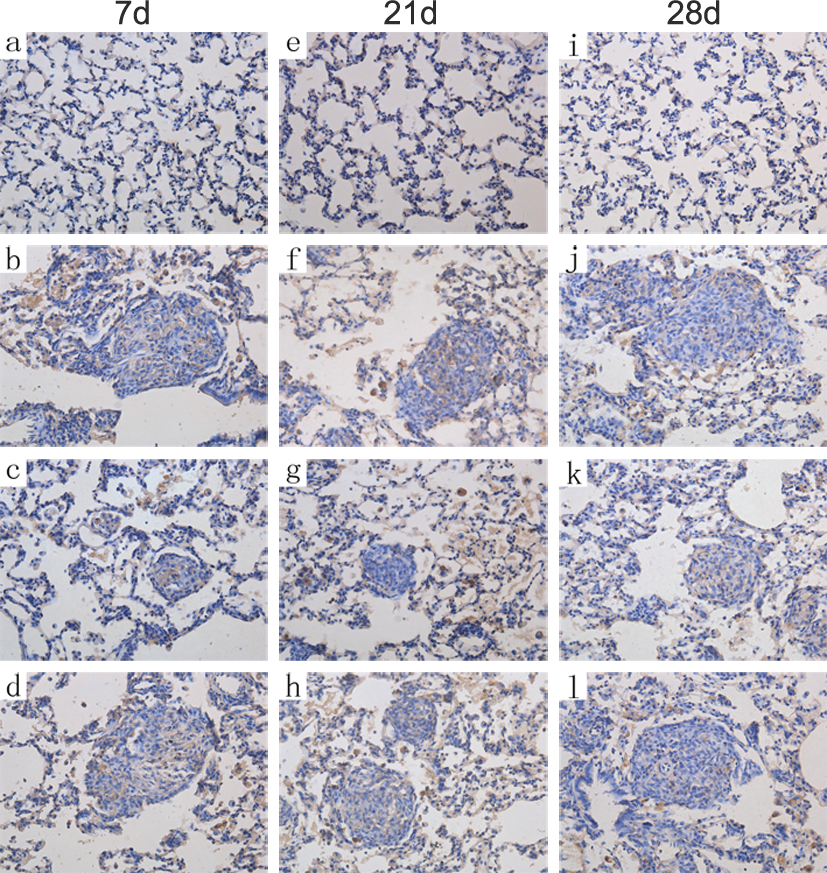

Supplement: Additional File 2 — Immunohistochemical staining for collagen I at each time point (× 400). 7d: a) saline control group, b) silica group, c) silica+Lv-shCD36, and d) silica+Lv-shCD36-NC; 21d: e) saline control group, f) silica group, g) silica+Lv-shCD36, and h) silica+Lv-shCD36-NC; 28d: i) saline control group, j) silica group, k) silica+Lv-shCD36, and l) silica+Lv-shCD36-NC. The expression of collagen I in the saline control group was negative at three time points. The expression of collagen I in the silica+Lv-shCD36 group was weaker than in the silica group and the silica+Lv-shCD36-NC group at three time points. [file 1465-9921-10-36-S2.tiff]

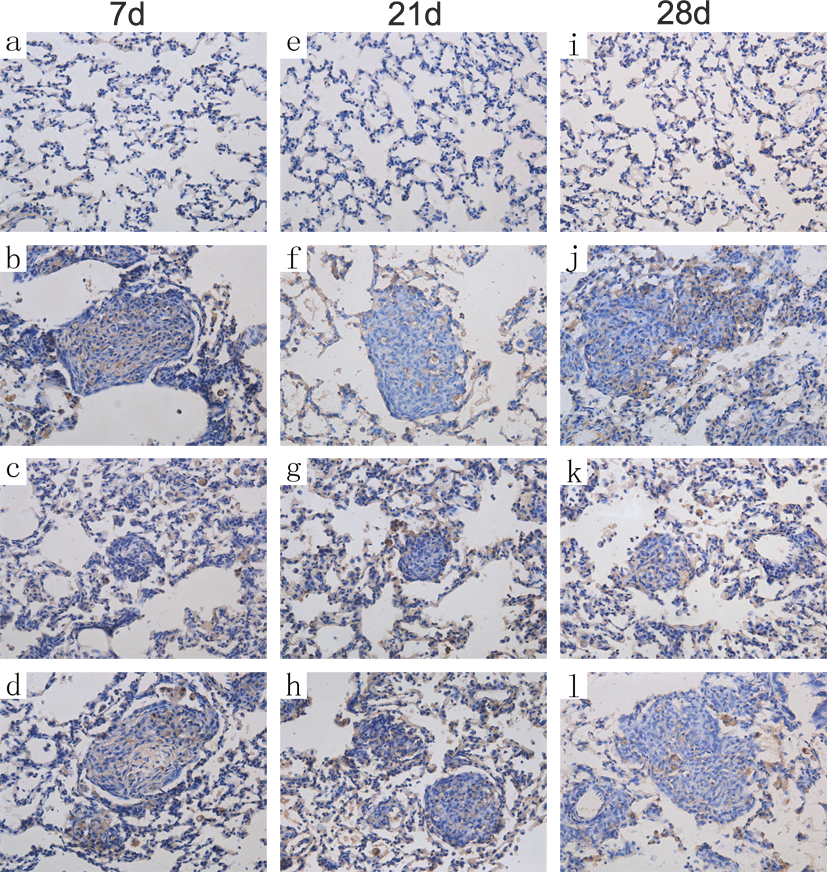

Supplement: Additional File 3 — Immunohistochemical staining for collagen III at each time point (× 400). 7d: a) saline control group, b) silica group, c) silica+Lv-shCD36, and d) silica+Lv-shCD36-NC; 21d: e) saline control group, f) silica group, g) silica+Lv-shCD36, and h) silica+Lv-shCD36-NC; 28d: i) saline control group, j) silica group, k) silica+Lv-shCD36, and l) silica+Lv-shCD36-NC. The expression of collagen I in the saline control group was negative at three time points. The expression of collagen III in the silica+Lv-shCD36 group was weaker than in the silica group and the silica+Lv-shCD36-NC group at three time points. [file 1465-9921-10-36-S3.tiff]
